# Isoquercitrin Attenuates Oxidative Liver Damage Through AMPK-YAP Signaling: An Integrative In Silico, In Vitro, and In Vivo Study

**DOI:** 10.3390/ijms26062717

**Published:** 2025-03-18

**Authors:** So-Hyun Kwon, Won-Yung Lee, Young Woo Kim, Kwang Suk Ko, Seon Been Bak, Sun-Dong Park

**Affiliations:** 1 School of Korean Medicine, Dongguk University, Gyeongju 38066, Republic of Korea; 2 College of Korean Medicine, Wonkwang University, Iksan 54538, Republic of Korea; 3 Department of Nutritional Science and Food Management, Ewha Womans University, Seoul 03760, Republic of Korea

**Keywords:** isoquercitrin, oxidative liver damage, hepatoprotective effects, LKB1/AMPK pathway, antioxidant activity

## Abstract

Isoquercitrin, a flavonoid glycoside found in various plants, has demonstrated antioxidant, anti-inflammatory, and anticancer properties. However, its hepatoprotective effects and underlying mechanisms against oxidative liver injury remain unclear. In this study, we evaluated the antioxidant and hepatoprotective effects of isoquercitrin using integrated in silico, in vitro, and in vivo approaches. HepG2 cells exposed to arachidonic acid (AA) and iron exhibited oxidative stress-induced apoptosis, which was significantly attenuated by isoquercitrin treatment, as evidenced by increased cell viability and reduced apoptosis-related protein alterations. Isoquercitrin decreased reactive oxygen species (ROS) generation and preserved mitochondrial function in a dose-dependent manner. Molecular docking and Western blot analyses revealed that isoquercitrin activates the LKB1/AMPK pathway, increasing phosphorylation of AMPK and its downstream target ACC, thereby modulating energy metabolism and reducing oxidative stress. This activation was LKB1 dependent, as confirmed in LKB1-deficient HeLa cells. Additionally, isoquercitrin modulated the YAP signaling pathway in hepatic cells. In vivo, isoquercitrin protected mice against carbon tetrachloride-induced liver injury, reducing serum ALT and AST levels and improving histopathological features. These findings suggest that isoquercitrin exerts hepatoprotective effects by activating the LKB1/AMPK pathway and modulating metabolic enzymes, highlighting its potential as a therapeutic agent against oxidative liver damage.

## 1. Introduction

The liver is one of the largest and most vital organs in the human body, playing a central role in regulating energy metabolism by producing and storing essential nutrients [1]. It performs multiple critical functions, such as detoxifying drugs and harmful substances, storing blood and various nutrients, and regulating the immune system [2]. Due to its central role in metabolism and detoxification, the liver is particularly susceptible to damage from oxidative stress. Oxidative stress arises when there is an imbalance between the production of reactive oxygen species (ROS) and the body’s antioxidant defenses. Excessive ROS can damage key cellular components—proteins, lipids, and DNA—leading to impaired liver function [3]. This oxidative damage is a common underlying mechanism in various liver diseases, including autoimmune, drug-induced, alcoholic, infectious, and congenital metabolic liver diseases [4,5,6]. Patients suffering from oxidative liver damage may exhibit symptoms such as fatigue, anorexia, indigestion, jaundice, and a tendency to bleed, underscoring the critical impact of oxidative stress on liver health. ROS are involved in a range of physiological and pathological processes. In the liver, acute exposure to severe oxidative stress results in hepatocellular death and inflammation [3]. ROS not only induce cellular damage but also influence gene expression in signal transduction pathways, affecting processes like cell growth and death [7]. Given the significant role of oxidative stress in liver pathology, developing effective strategies to protect hepatocytes and prevent liver diseases is of paramount importance.

Natural products, especially flavonoids, have garnered significant attention for their potential to prevent oxidative liver damage [8]. Flavonoids are a diverse group of plant secondary metabolites known for their antioxidant, anti-inflammatory, and hepatoprotective properties. They exert protective effects by scavenging free radicals, chelating metal ions, and modulating antioxidant enzyme activities, thereby mitigating oxidative stress and cellular damage in the liver [9]. Among these flavonoids, isoquercitrin—a quercetin glycoside found in various medicinal herbs, fruits, and vegetables—has demonstrated a range of biological activities, including antioxidant, anti-inflammatory, and anticancer effects [10,11]. Isoquercitrin has been reported to reduce ROS levels, inhibit lipid peroxidation, and protect against oxidative stress-induced cellular damage [12]. In particular, studies have shown that it induces apoptosis and autophagy in hepatocellular carcinoma cells via signaling pathways [12]; exhibits antioxidant activity, including reduced ROS levels and the inhibition of in vivo and in vitro lipid peroxidation [13]; and promotes neuroprotection and neurite elongation [14]. However, the effects of isoquercitrin on oxidative liver injury and its underlying molecular mechanisms are not yet fully understood.

Recent advances have highlighted the importance of integrating in silico analyses with in vitro and in vivo experiments to fully understand the therapeutic potential of natural products [15]. In silico methods, such as molecular docking, enable the prediction of interactions between bioactive compounds and target proteins, providing insights into their mechanisms of action [16,17]. For instance, kaempferol was shown to stimulate autophagy and regulate ferroptosis under oxidative stress through AMP-activated protein kinase (AMPK) signaling, with molecular docking confirming its binding to key proteins involved in these pathways [18]. Similarly, an integrative approach combining in silico analysis and experimental validation was used to uncover the antioxidant properties of Bupleuri Radix and its active compounds [19]. These studies demonstrate that combining computational modeling with experimental studies enhances our understanding of how natural products exert their biological effects.

In this study, we conducted a comprehensive investigation integrating in silico, in vitro, and in vivo experiments to evaluate the antioxidant and hepatoprotective effects of isoquercitrin against oxidative liver damage. We first observed that isoquercitrin exhibited a protective effect on hepatocytes subjected to oxidative stress induced by the arachidonic acid (AA) and iron model. Subsequently, we confirmed that isoquercitrin effectively inhibited ROS production in these cells. Molecular docking studies were performed to assess the stable binding affinity of isoquercitrin to key proteins within the AMP-activated protein kinase (AMPK) pathway, and we examined its impact on the activation of AMPK and acetyl-CoA carboxylase (ACC). Similar analyses were conducted on the Yes-associated protein (YAP) pathway to explore additional mechanisms underlying its hepatoprotective effects. Finally, we evaluated whether the oral administration of isoquercitrin could prevent liver toxicity using in vivo models. Through this integrated approach, our study aims to elucidate the potential of isoquercitrin as a therapeutic agent for protecting against oxidative liver injury.

## 2. Results

### 2.1. Effect of Isoquercitrin on Mitochondrial Damage Induced by Hepatotoxicity

**Figure 1:**
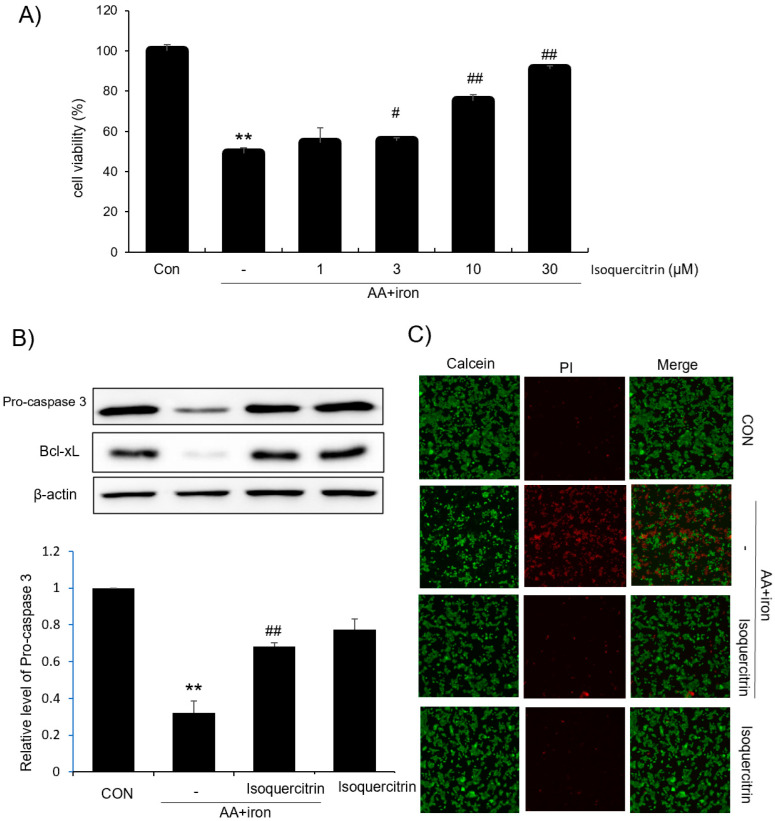
Isoquercitrin enhances cell viability and modulates apoptosis-related proteins. (**A**) Cell viability of HepG2 cells treated with isoquercitrin at varying concentrations. (**B**) Western blot analysis of apoptosis-related proteins in HepG2 cells, including procaspase-3, and Bcl-xL. β-actin was used as a loading control. (**C**) Representative fluorescence images showing the effect of isoquercitrin treatment on cell death (×200). Data are presented as the average of repeated samples (*n* = 3), with error bars representing S.D. (vs. control ** *p* < 0.01; vs. AA+iron; # *p* < 0.05, ## *p* < 0.01).

AA treatment induces oxidative stress and apoptosis in HepG2 cells, and iron treatment increases AA-induced toxicity. In this study, an MTT assay was performed to observe the inhibitory effect of isoquercitrin on apoptosis induced by AA+iron in HepG2 cells. As a result, the cell viability decreased by AA+iron showed a tendency to increase in an isoquercitrin dose-dependent manner, and the most effective cell protection was shown at concentrations of 10 and 30 μg/mL (Figure 1A). Immunoblotting of apoptosis-related proteins was performed following the MTT assay. Isoquercitrin significantly inhibited the changes in Bcl-xL and procaspase-3 induced by AA+iron treatment in HepG2 cells (Figure 1B). Moreover, isoquercitrin also inhibited the activity of caspase 3 [i.e., control, 1.0; AA+iron, 1.38; AA+iron+ isoquercitrin, 1.15 (fold change vs. control)]. These results indicate that cell death by AA+iron in HepG2 cells is associated with the induction of apoptosis, and isoquercitrin has a cytoprotective effect against AA+iron-induced apoptosis. Next, the cytoprotective effect of isoquercitrin on AA+iron-induced hepatotoxicity was confirmed again using fluorescence microscopy, which uses an ultraviolet light source to observe intracellular fluorescent materials. After the co-treatment of HepG2 cells with 30 μg/mL isoquercitrin and AA+iron, PI staining was increased compared with that in the other groups, indicating intense fluorescence and potential cell membrane damage. These results suggest that isoquercitrin treatment has a potential therapeutic role in protecting liver cells and alleviating AA+iron-induced hepatotoxicity (Figure 1C).

### 2.2. Effect of Isoquercitrin on Mitochondrial Damage

**Figure 2:**
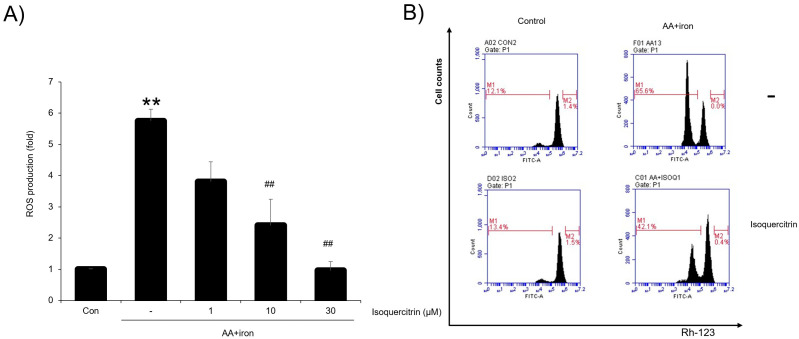
Isoquercitrin reduces AA+iron-induced ROS production and preserves mitochondrial function. (**A**) Effect of isoquercitrin on reactive oxygen species (ROS) production in AA+iron-treated HepG2 cells. ROS levels were significantly elevated in the AA+iron-treated group (−) compared to those in the control group (Con). Isoquercitrin treatment at concentrations of 1, 10, and 30 μM effectively reduced ROS production in a dose-dependent manner. (**B**) Flow cytometry analysis of mitochondrial membrane potential disruption, an indicator of mitochondrial dysfunction. Data are presented as the average of repeated samples (*n* = 4), with error bars representing S.D. (vs. control ** *p* < 0.01; vs. AA+iron; ## *p* < 0.01).

The effects of isoquercitrin on oxidative stress and cell differentiation under stress conditions were evaluated. To measure ROS scavenging activity, a DCFH-DA assay was performed using HepG2 cells. HepG2 cells were seeded in 96-well plates and cultured. The cells were treated with isoquercitrin at concentrations of 1, 10, and 30 μg/mL, followed by treatment with 5 μM iron + 10 μM AA for 3 h. To measure the amount of ROS, 10 μM DCFH-DA was added for 1 h. Before sampling, 2′,7′-dichlorofluorescein diacetate (DCFH-DA) reagent was incubated for 1 h, and then the fluorescence level of DCF was analyzed using an ELISA microplate reader (Figure 2A). The results showed that isoquercitrin significantly reduced ROS generation in a dose-dependent manner, indicating potent antioxidant activity. In particular, high concentrations of isoquercitrin effectively counteracted the increase in ROS levels induced by AA+iron treatment. Furthermore, the effects of isoquercitrin on cell differentiation and mitochondrial function were evaluated using flow cytometry (Figure 2B). In the control group, the percentage of cells in the M1 stage was 12.1%, but it dramatically increased to 65.6% when treated with AA+iron, indicating cell cycle arrest due to significant oxidative stress. However, cells treated with isoquercitrin alone maintained a low percentage of cells in the M1 stage at 13.4%, similar to the control group. Isoquercitrin treatment also prevented the collapse of mitochondrial membrane potential observed in the AA+iron group, as shown by flow cytometry analysis. These findings demonstrate that isoquercitrin effectively alleviates oxidative stress and mitochondrial dysfunction while preserving normal cell differentiation even under stress conditions.

### 2.3. Activation of the AMPK Pathway by Isoquercitrin

**Figure 3:**
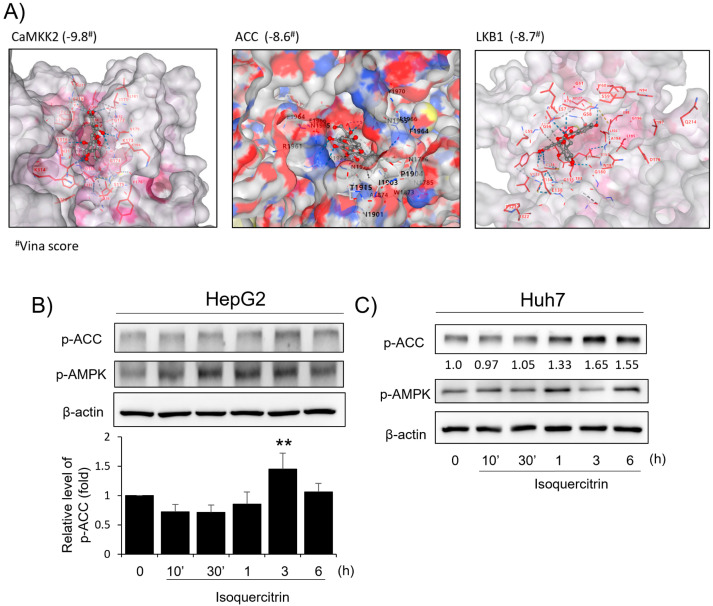
Molecular docking and effects of isoquercitrin on the antioxidant AMPK pathway. (**A**) Molecular docking analysis of isoquercitrin with CaMKK2, ACC, and LKB1. Docking results highlight the binding interactions between isoquercitrin and the active sites of the respective proteins. Western blot analysis of P-ACC and P-AMPK protein expression in HepG2 cells treated with isoquercitrin at various time points (0, 10′, 30′, 1 h, 3 h, and 6 h) in HepG2 (**B**) and Huh7 (**C**) cells. Data are presented as the average of repeated samples (*n* = 3), with error bars representing S.D. (vs. control ** *p* < 0.01). The number below the image indicates the relative intensity of the blot (fold change vs. control).

To explore the primary mechanisms underlying the antioxidant effects of isoquercitrin, we first examined its impact on the AMPK signaling pathway. Although antioxidant functions are generally associated with Nrf2 signaling, previous findings showed that AA+iron is closely related to AMPK as an anti-oxidative signaling pathway. Molecular docking analysis was performed to predict the interaction between isoquercitrin and key proteins in the AMPK pathway (Figure 3A). Isoquercitrin exhibited strong binding affinities to AMPK-related proteins, with docking scores of −9.8, −8.6, and −8.7, suggesting its potential to directly interact with and modulate these targets. Subsequently, Western blot analysis was conducted to evaluate the effects of isoquercitrin on the activation of the AMPK pathway (Figure 3B). Isoquercitrin treatment significantly increased the phosphorylation levels of AMPK (p-AMPK) and its downstream target ACC (p-ACC), indicating activation of the pathway. The phosphorylation levels peaked at 3 h post-treatment, demonstrating a time-dependent effect. Consistent results were observed in Huh7 cells, further confirming the ability of isoquercitrin to activate the AMPK pathway. These findings highlight the role of the AMPK pathway as a key mechanism through which isoquercitrin exerts its protective effects against oxidative stress.

### 2.4. LKB1 Dependency of Isoquercitrin Effects on the AMPK Pathway

**Figure 4:**
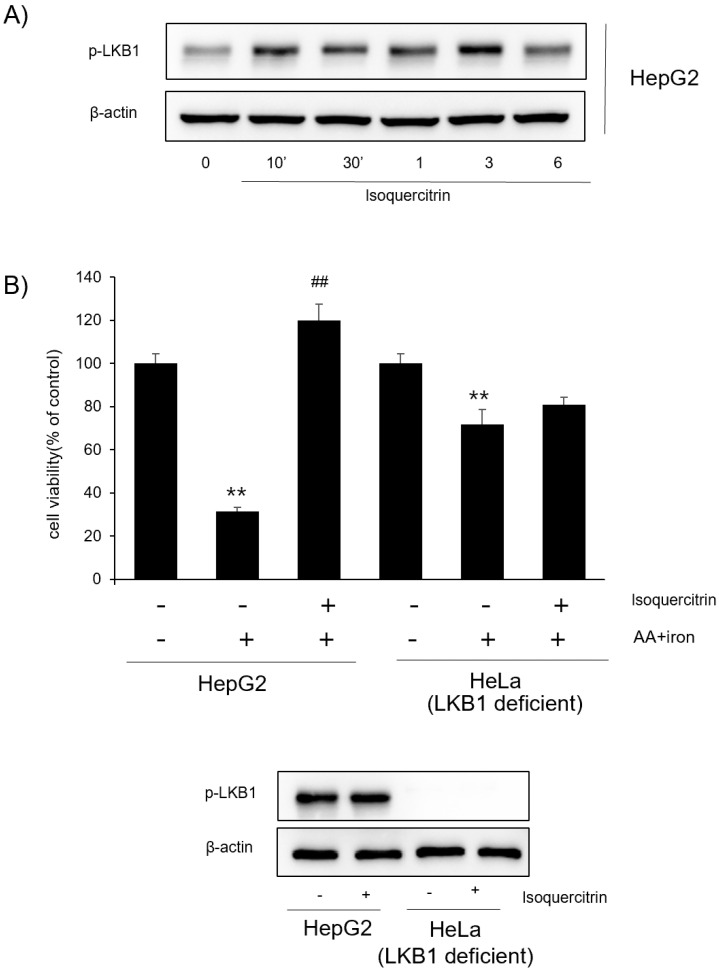
Effects of isoquercitrin on LKB1 phosphorylation. (**A**) Western blot analysis showing the time-dependent activation of LKB1 in HepG2 cells treated with isoquercitrin at various time points (0, 10′, 30′, 1 h, 3 h, and 6 h). (**B**) MTT assay. HepG2 and HeLa cells were treated with AA+iron with or without isoquercitrin (30 μM). Data are presented as the average of repeated samples (*n* = 3), with error bars representing S.D. (vs. control ** *p* < 0.01; vs. AA+iron; ## *p* < 0.01).

To further elucidate the upstream signaling mechanism involved in isoquercitrin-induced AMPK activation, we investigated the role of LKB1, a well-known upstream kinase of AMPK. Western blot analysis revealed that isoquercitrin treatment significantly increased the phosphorylation levels of LKB1 over time, with peak activation observed at 3 h post-treatment (Figure 4A). This indicates that LKB1 may play a critical role in mediating isoquercitrin’s effects on the AMPK pathway. To confirm the dependency of isoquercitrin’s effects on LKB1, we utilized HeLa cells, which lack functional LKB1 expression (Figure 4B). In HeLa cells, isoquercitrin failed to induce AMPK phosphorylation, demonstrating a lack of isoquercitrin-induced effects in the absence of LKB1. These findings suggest that isoquercitrin-mediated AMPK activation occurs in an LKB1-dependent manner and highlight the importance of the LKB1/AMPK axis in the antioxidant and protective mechanisms of isoquercitrin.

### 2.5. YAP Pathway Activation by Isoquercitrin in HepG2 and Huh7 Cells

**Figure 5:**
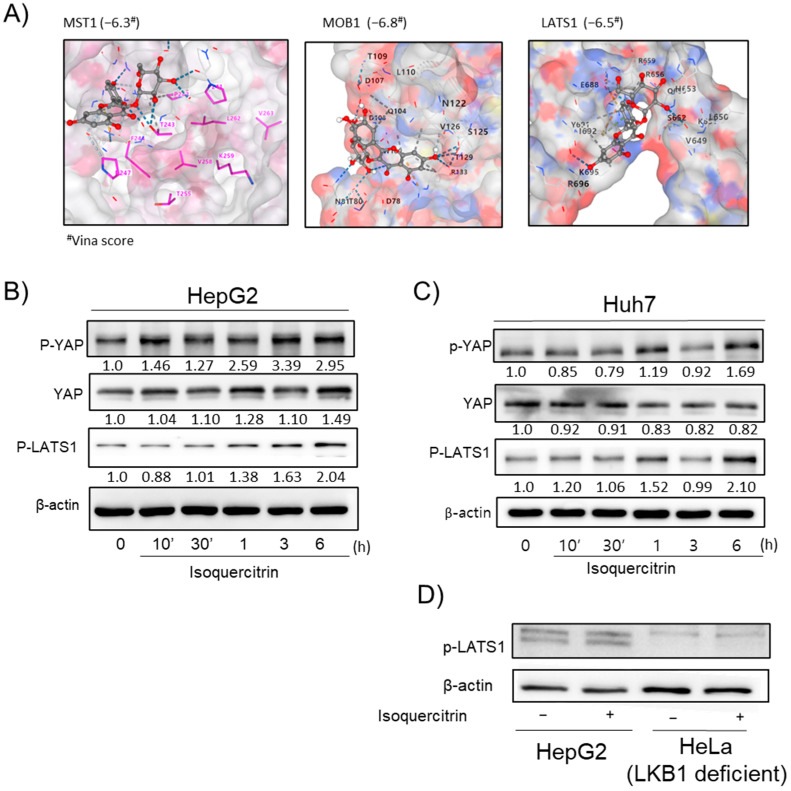
Effects of isoquercitrin on the YAP pathway. (**A**) Molecular docking analysis of isoquercitrin with YAP and LATS1. Docking results highlight the binding interactions between isoquercitrin and the active sites of the respective proteins. Western blot analysis of P-YAP and P-LATS1 protein expression in HepG2 cells treated with isoquercitrin at various time points (0, 10′, 30′, 1 h, 3 h, and 6 h) in HepG2 (**B**) and Huh7 (**C**) cells. (**D**) Effect of isoquercitrin on LKB1-deficient HeLa cells. The number below the image indicates the relative intensity of the blot (fold change vs. control).

To explore the involvement of the YAP signaling pathway in isoquercitrin’s mechanism of action, we conducted molecular docking analysis with YAP pathway-related proteins, including MST1, MOB1, and LATS1 (Figure 5A). Isoquercitrin exhibited binding affinities with docking scores of −6.3, −6.8, and −6.5, respectively, suggesting its potential interactions with key regulators of the YAP pathway. Next, the functional effects of isoquercitrin on YAP activation were evaluated in HepG2 cells. Western blot analysis showed that isoquercitrin enhanced YAP activity, as evidenced by increased phosphorylation levels of key downstream proteins in the pathway (Figure 5B). Similar results were observed in Huh7 cells, where isoquercitrin treatment also led to a pronounced activation of the YAP pathway (Figure 5C). To further confirm the dependency of isoquercitrin’s effects on YAP pathway components, experiments were performed in HeLa cells. In HeLa cells, isoquercitrin failed to activate YAP signaling, indicating that its effects on the pathway are context-dependent and require specific components of the YAP signaling cascade. These results suggest that isoquercitrin modulates the YAP pathway in a cell-specific manner, with functional activation observed in HepG2 and Huh7 cells but not in HeLa cells.

### 2.6. Protective Effects of Isoquercitrin on CCl4-Induced Liver Injury in Mice

**Figure 6:**
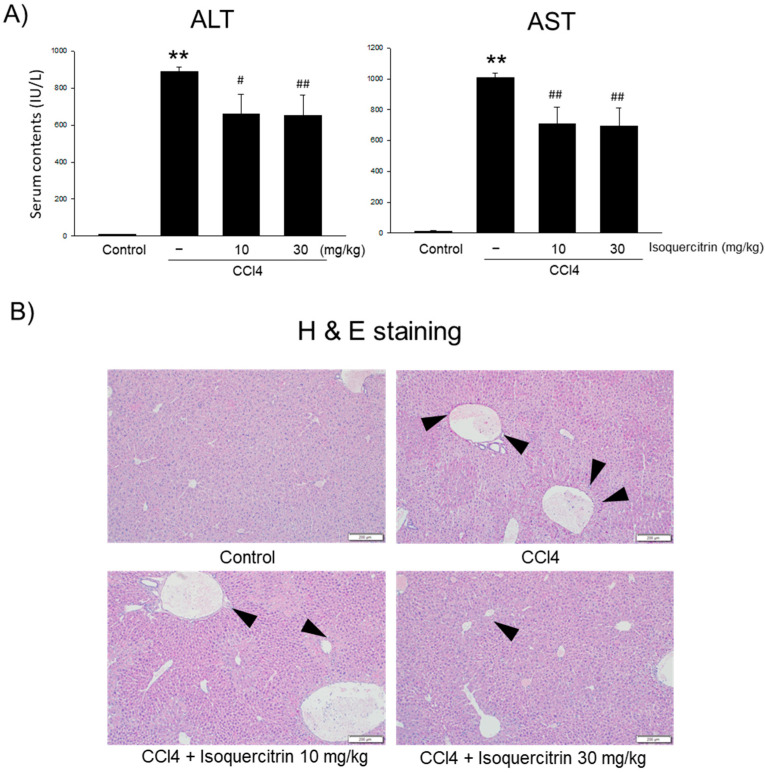
Protective effects of isoquercitrin on serum biochemical markers and liver histopathology in CCl4-induced liver damage. (**A**) Serum ALT and AST levels in experimental groups. CCl4 significantly elevated ALT and AST levels compared to those in the control group. The data represent the mean ± S.E.M. (** *p* < 0.01 between the vehicle control; ## *p* < 0.01, # *p* < 0.05 between CCl4 treatments). (**B**) Representative histological analysis of liver tissues stained with H&E (bar = 200 μm). Arrows indicate hepatic degeneration.

To investigate whether isoquercitrin exerts protective effects in vivo, we utilized a CCl4-induced liver injury mice model and evaluated the outcomes following the oral administration of isoquercitrin. Serum levels of ALT and AST, markers of liver damage, were significantly elevated in the CCl4-treated group compared to those in the control group (Figure 6A). However, isoquercitrin treatment significantly reduced ALT and AST levels in a dose-dependent manner, indicating its ability to protect against CCl4-induced liver injury. Additionally, histopathological analysis of liver tissues was performed using H&E staining (Figure 6B). In the CCl4-treated group, severe pathological changes were observed, including hepatocyte necrosis, inflammatory cell infiltration, and structural disruption. In contrast, the isoquercitrin-treated groups exhibited markedly reduced pathological damage, with preserved liver architecture and decreased necrosis and inflammation compared to the CCl4-only group. These findings demonstrate that isoquercitrin effectively mitigates CCl4-induced liver damage both biochemically and histologically, supporting its therapeutic potential for liver protection.

## 3. Discussion

In this study, we evaluated the hepatoprotective and antioxidant effects of isoquercitrin and investigated its underlying molecular mechanisms. We found that isoquercitrin significantly enhanced cell viability in HepG2 cells exposed to AA+iron-induced oxidative stress (Figure 1A). The cell viability increased in a dose-dependent manner, with the most effective protection observed at concentrations of 10 and 30 μg/mL. We also observed that isoquercitrin inhibited the decrease in apoptosis-related proteins Bcl-xL and procaspase-3 induced by AA+iron treatment (Figure 1B), indicating its anti-apoptotic effects. Moreover, it is valuable to analyze the expression of the cleaved form of caspase 3 as well as procaspase-3 in the future. Fluorescence microscopy further confirmed that isoquercitrin reduced PI staining intensity compared to that in the AA+iron-treated group, suggesting that it prevented cell membrane damage and apoptosis (Figure 1C). These findings suggest that isoquercitrin exerts cytoprotective effects by inhibiting apoptosis in hepatocytes under oxidative stress conditions. Also, we demonstrated that isoquercitrin effectively reduced ROS generation in a dose-dependent manner (Figure 2A). The DCFH-DA assay showed that isoquercitrin significantly decreased ROS levels elevated by AA+iron treatment, demonstrating its potent antioxidant activity. Flow cytometry analysis revealed that isoquercitrin prevented the collapse of mitochondrial membrane potential induced by oxidative stress (Figure 2B). By preserving mitochondrial function, isoquercitrin helps maintain cellular energy metabolism and prevent cell death.

A key finding was the activation of the AMPK pathway by isoquercitrin. Molecular docking analysis indicated strong binding affinities between isoquercitrin and AMPK-related proteins, such as AMPK, CaMKK2, ACC, and LKB1 (Figure 3A). This suggests that isoquercitrin may directly interact with these proteins to modulate their activity. Western blot analysis showed that isoquercitrin increased the phosphorylation levels of AMPK and its downstream target ACC in a time-dependent manner, peaking at 3 h post-treatment (Figure 3B). This activation was consistent in both HepG2 and Huh7 cells, indicating a broader applicability. We further explored the role of LKB1 in isoquercitrin-induced AMPK activation. Our results showed that isoquercitrin increased the phosphorylation of LKB1 (Figure 4A), suggesting that LKB1 is involved in the upstream regulation of AMPK activation by isoquercitrin. In LKB1-deficient HeLa cells, isoquercitrin failed to induce AMPK phosphorylation (Figure 4B), confirming that LKB1 is essential for this pathway. These findings align with previous studies indicating that LKB1 is a primary activator of AMPK [20,21]. Activation of the LKB1/AMPK pathway by isoquercitrin has significant implications for energy metabolism. AMPK activation inhibits ACC, reducing fatty acid synthesis and promoting fatty acid oxidation [22]. By regulating these metabolic enzymes, isoquercitrin helps restore energy homeostasis and may prevent metabolic disorders [12,23]. In fact, the molecular manipulation of AMPK in the liver cells is important in this study. Therefore, some molecular tools such as siRNA would be recommended in the future for similar studies with HeLa cells. Our study supports the notion that isoquercitrin can improve energy metabolism by activating the LKB1/AMPK pathway.

In addition to our findings, several independent studies have reported that isoquercitrin can activate or modulate the AMPK signaling pathway in diverse cellular contexts. For instance, a research demonstrated that isoquercitrin triggers apoptosis in bladder cancer cells via activation of the AMPK pathway, suggesting that isoquercitrin’s effects on energy metabolism are not restricted to hepatocytes [24]. Similarly, isoquercitrin has been shown to induce both apoptosis and autophagy in hepatocellular carcinoma cells by modulating the AMPK/mTOR/p70S6K signaling axis, further supporting its role as an AMPK activator [12]. Moreover, recent evidence indicates that isoquercitrin may enhance oxidative stress and promote ferroptosis in nasopharyngeal carcinoma cells via modulation of AMPK/NF-κB signaling [11]. Collectively, these reports corroborate our observation that isoquercitrin activates the AMPK pathway and suggest that the compound’s beneficial effects on lipid metabolism and cell survival may be mediated, at least in part, by its modulation of AMPK signaling.

We also investigated the modulation of the YAP signaling pathway by isoquercitrin. Molecular docking suggested potential interactions between isoquercitrin and YAP pathway proteins, such as MST1, MOB1, and LATS1 (Figure 5A). Western blot analysis showed that isoquercitrin enhanced YAP activity in HepG2 and Huh7 cells (Figure 5B,C), but not in HeLa cells, indicating a cell-specific effect. The modulation of the YAP pathway may contribute to isoquercitrin’s hepatoprotective effects by influencing cell proliferation and survival pathways.

In our in vivo studies, we evaluated the protective effects of isoquercitrin on CCl_4_-induced liver injury in mice. The oral administration of isoquercitrin significantly reduced serum ALT and AST levels compared to those in the CCl_4_-only group (Figure 6A), indicating decreased liver damage. Histopathological analysis confirmed that isoquercitrin mitigated liver tissue damage, preserving hepatic architecture and reducing necrosis and inflammation (Figure 6B). Although the AMPK level in the liver in important, we could not confirm changes in protein expression in the liver, which need to be clarified in the future. These findings demonstrate that isoquercitrin’s hepatoprotective effects observed in vitro translate to in vivo models.

We employed a comprehensive strategy integrating molecular docking analyses with both in vitro and in vivo experiments to explore the effects of isoquercitrin. However, our study is not without limitations: Although our in vitro data and molecular docking analysis indicate a direct interaction between isoquercitrin and AMPK-related proteins, further validation using direct binding assays and primary hepatocyte models is needed. In future studies, it will be interesting to (1) perform in vivo experiments using NAFLD animal models to evaluate the long-term effects of isoquercitrin on hepatic lipid metabolism and (2) explore additional molecular mechanisms—such as potential crosstalk with mTOR and YAP signaling pathways—that may further explain the cytoprotective properties of isoquercitrin. These efforts will help to clarify the detailed molecular interactions underlying isoquercitrin-mediated AMPK activation and strengthen its potential application in the prevention of metabolic disorders.

## 4. Materials and Methods

### 4.1. Reagents

Isoquercitrin was purchased from Sigma-Aldrich. Some antibodies against anti-phospho AMP-activated protein kinase (p-AMPK, #2535), anti-AMPK, anti-phospho acetyl-CoA carboxylase (p-ACC, #3661), anti-poly (ADP-ribose) polymerase (PARP, #9542), anti-procaspase 3, anti-phospho liver kinase B1 (p-LKB1, #3482), liver kinase B1 (LKB1, #3050), and Bcl-xL were purchased from Cell Signaling Technology (Beverly, MA, USA), and anti-mouse and anti-rabbit antibodies were purchased from Santa Cruz Biotechnology, Inc. (Santa Cruz, CA, USA). Additionally, arachidonic acid (AA) and rhodamine123 (Rho123) were purchased from Calbiochem (San Diego, CA, USA), and 2′,7′-dichlorodihydrofluorescin diacetate (H2DCF-DA), ferric nitrilotriacetic acid (Fe-NTA), and 3-(4, 5-dimethylthiazol-2-yl)-2, 5-diphenyltetrazoleum bromide (MTT) were purchased from Sigma (St. Louis, MO, USA).

### 4.2. Cell Culture and Treatment

HepG2 cells, a human-derived liver parenchymal cell line, used in the experiment, as well as Hela cells and Huh7 cells, were purchased from the American Type Culture Collection (ATCC, Rockville, MD, USA). All cells were cultured at 37 °C in a medium containing 10% bovine fetal serum (FBS), 100 μg/mL penicillin, and 1 mL Normocin in Dulbecco’s modified Eagle’s medium (DMEM) and 5% CO_2_ in an incubator [19].

Afterward, the cells were dispensed onto plates suitable for each experiment and incubated for 12 h with the above DMEM, which was then replaced with FBS-free and serum-deficient media for 12 h. Then, 1–30 μg/mL of isoquercitrin and 10 μM AA were added simultaneously for 12 h, followed by 5 μM iron for 1–6 h. All cells in the experiment were tested at 80–90% confluence.

### 4.3. Cell Viability Test (MTT Assay)

HepG2 cells were seeded in a 48-well plate at 1 × 10^5^ cells/well and cultured for 24 h. After treatment with 0, 1, 3, 10, 30, and 100 µg/mL of isoquercitrin and 10 µM AA for 12 h, 5 µM iron was further added for 3 h. 3-(4, 5-dimethylthiazol-2-yl)-2, 5-diphenyltetrazoleum bromide (MTT) reagent was added at 0.1 mg/mL and reacted at 37 °C for 1 h. The generated formazan crystals were dissolved with dimethyl sulfoxide (DMSO), and the absorbance was measured at 570 nm using a microplate measuring device (Tecan Infinite^®^ M200 PRO, Baldwin Park, CA, USA) [19].

### 4.4. Immunoblot Analysis

Cell lysates were prepared using lysis buffer (Thermo, Rockford, IL, USA), and were also homogenized with a sonicator (Sonics, USA). After centrifugation at 15,000× *g* for 30 min, only the supernatant was collected, and the protein was quantified using a bicinchoninic acid (BCA) protein assay kit (Pierce, Rockford, IL, USA). Next, the proteins extracted from the cells were separated by size by 10% SDS-PAGE and then electrotransferred from the acrylamide gel to a nitrocellulose membrane. Afterward, the nitrocellulose membrane was treated overnight at 4 °C with antibodies appropriate for each protein. HRP-linked anti-rabbit or anti-mouse secondary antibodies were used, and color was developed using ECL chemiluminescence detection reagents and then detected using a Chemidoc image analyzer (Vilber Lourmat, France) previously described [19].

### 4.5. Measurement of Intracellular ROS

To measure the intracellular antioxidant activity of isoquercitrin, ROS was quantified using 2′,7′-dichlorofluorescein diacetate (DCFH-DA). HepG2 cells were treated with 10 µM DCFH-DA for 1 h at 37 °C to induce cell response, which was then oxidized to 2′,7′-dichlorofluorescein (DCF) by ROS and exhibited high fluorescence. The fluorescence level of DCF was analyzed using an ELISA microplate reader [19].

### 4.6. Measurement of Mitochondrial Membrane Permeability (MMP)

To observe the mitochondrial protective effect of isoquercitrin, MMP was evaluated using flow cytometry after staining with rhodamine 123 (Rh123), a mitochondrion-specific green fluorescent dye. HepG2 cells were seeded at a density of 1 × 10^5^ per well in 6-well plates for 24 h and then cultured in serum-free MEM for 12 h. After that, 30 µg/mL of isoquercitrin and 10 µM AA were added and reacted for 12 h, followed by 5 µM iron for 3 h and staining with 0.05 µg/mL Rh123 for 1 h. Cells were harvested using trypsin and resuspended in PBS, and MMP changes were measured using a flow cytometer (FACS, Partec, Münster, Germany). MMP was observed by recording a population of 10,000 cells [19].

### 4.7. Fluorescence Microscopy

HepG2 cells were cultured in 6-well plates at 1 × 10^6^ cells/well. Isoquercitrin 30 µg/mL was added with 10 µM AA for 12 h, and then 5 µM iron was reacted for 3 h. Then, calcein and PI were added at a concentration of 0.5 µM for 1 h.

### 4.8. Molecular Docking

Molecular docking studies were performed to predict the potential binding of isoquercitrin to key regulatory proteins in the AMPK and YAP signaling pathways using the CB-Dock2 online docking server (https://cadd.labshare.cn/cb-dock2, accessed on 8 July 2024) [25]. The three-dimensional structure of isoquercitrin (PubChem CID: 5280804) was obtained from the PubChem database in SDF format and directly uploaded to the CB-Dock2 server without further manual preprocessing. The crystal structures of the target proteins—CaMKK2 (PDB ID: 5UYJ), ACC (3H0J), AMPK (4CFE), LKB1 (2WTK), MST1 (3COM), MOB1 (1PI1), and LATS1 (5B5W)—were downloaded from the Protein Data Bank (PDB) in PDB format. For each docking run, the ligand (isoquercitrin) and protein structures were uploaded to the CB-Dock2 server, which automatically detects the binding pockets and performs docking calculations using default parameters. The server outputs a list of binding poses ranked by predicted binding energies (docking scores). The top-ranking poses were selected for subsequent visualization and analysis.

### 4.9. Statistical Treatment

The results of this study were expressed as the mean ± standard error (mean ± S.E.M.) of the results of experiments repeated at least three times. The comparison of each treatment group was performed using one-way analysis of variance (ANOVA) or Student’s *t*-test was used to verify statistical significance (*p* < 0.05 or *p* < 0.01)

## 5. Conclusions

Isoquercitrin was confirmed to have a significant effect on energy metabolism and oxidative stress through the AMPK/LKB1 pathway in HepG2 cells. The activation of AMPK through this pathway maximized the antioxidant effect by altering the metabolic balance of cells and effectively attenuating liver damage from oxidative stress induced by AA and iron, confirming the potential liver-protective effect on oxidative stress in the prevention and treatment of metabolic diseases. Therefore, this study suggests that isoquercitrin has considerable potential to be developed as a natural hepatoprotective agent.

## Data Availability

The data presented in this study are available on request from the corresponding author. The data are not publicly available due to ethical restrictions.

## References

[1] Rui L. (2014). Energy Metabolism in the Liver. Compr. Physiol..

[2] Stravitz R.T., Lee W.M. (2019). Acute Liver Failure. Lancet.

[3] Cichoż-Lach H., Michalak A. (2014). Oxidative Stress as a Crucial Factor in Liver Diseases. World J. Gastroenterol. WJG.

[4] Roth A.D., Lee M.-Y. (2017). Idiosyncratic Drug-Induced Liver Injury (Idili): Potential Mechanisms and Predictive Assays. Biomed Res. Int..

[5] Stine J.G., Chalasani N.P. (2017). Drug Hepatotoxicity: Environmental Factors. Clin. Liver Dis..

[6] Lee W.M. (2013). Drug-Induced Acute Liver Failure. Clin. Liver Dis..

[7] Lee J., Giordano S., Zhang J. (2012). Autophagy, Mitochondria and Oxidative Stress: Cross-Talk and Redox Signalling. Biochem. J..

[8] Van De Wier B., Koek G.H., Bast A., Haenen G.R. (2017). The Potential of Flavonoids in the Treatment of Non-Alcoholic Fatty Liver Disease. Crit. Rev. Food Sci. Nutr..

[9] Gajender, Mazumder A., Sharma A., Azad M.A. (2023). A Comprehensive Review of the Pharmacological Importance of Dietary Flavonoids as Hepatoprotective Agents. Evid.-Based Complement. Altern. Med..

[10] Valentová K., Vrba J., Bancířová M., Ulrichová J., Křen V. (2014). Isoquercitrin: Pharmacology, Toxicology, and Metabolism. Food Chem. Toxicol..

[11] Luo X., Gong Y., Jiang Q., Wang Q., Li S., Liu L. (2024). Isoquercitrin Promotes Ferroptosis and Oxidative Stress in Nasopharyngeal Carcinoma Via the Ampk/Nf-κB Pathway. J. Biochem. Mol. Toxicol..

[12] Shui L., Wang W., Xie M., Ye B., Li X., Liu Y., Zheng M. (2020). Isoquercitrin Induces Apoptosis and Autophagy in Hepatocellular Carcinoma Cells Via Ampk/Mtor/P70s6k Signaling Pathway. Aging.

[13] Li R., Yuan C., Dong C., Shuang S., Choi M.M. (2011). In Vivo Antioxidative Effect of Isoquercitrin on Cadmium-Induced Oxidative Damage to Mouse Liver and Kidney. Naunyn-Schmiedeberg’s Arch. Pharmacol..

[14] Jung S.H., Kim B.J., Lee E.H., Osborne N.N. (2010). Isoquercitrin Is the Most Effective Antioxidant in the Plant Thuja Orientalis and Able to Counteract Oxidative-Induced Damage to a Transformed Cell Line (Rgc-5 Cells). Neurochem. Int..

[15] Peres R.B., Fiuza L.F.d.A., da Silva P.B., Batista M.M., Camillo F.d.C., Marques A.M., Brito L.D.C., Figueiredo M.R., Soeiro M.D.N. (2021). In Vitro Phenotypic Activity and in Silico Analysis of Natural Products from Brazilian Biodiversity on Trypanosoma Cruzi. Molecules.

[16] Jiaxin Z., Xinhao Z., Chaofeng Z., Wangning Z., Jiangwei T. (2024). Research Progress on New Techniques and Methods for Identifying Active Ingredients in Traditional Chinese Medicine. Chin. J. Nat. Med..

[17] Martorana A., Perricone U., Lauria A. (2016). The Repurposing of Old Drugs or Unsuccessful Lead Compounds by in Silico Approaches: New Advances and Perspectives. Curr. Top. Med. Chem..

[18] Wang X., Yang Y., An Y., Fang G. (2019). The Mechanism of Anticancer Action and Potential Clinical Use of Kaempferol in the Treatment of Breast Cancer. Biomed. Pharmacother..

[19] Bak S.B., Song Y.R., Bae S.J., Lee W.Y., Kim Y.W. (2023). Integrative Approach to Uncover Antioxidant Properties of Bupleuri Radix and Its Active Compounds: Multiscale Interactome-Level Analysis with Experimental Validation. Free Radic. Biol. Med..

[20] Xie W., Wang M., Chen C., Zhang X., Melzig M.F. (2016). Hepatoprotective Effect of Isoquercitrin against Acetaminophen-Induced Liver Injury. Life Sci..

[21] Steinberg G.R., Hardie D.G. (2023). New Insights into Activation and Function of the Ampk. Nat. Rev. Mol. Cell Biol..

[22] Wu X., Xu J., Cai Y., Yang Y., Liu Y., Cao S. (2021). Cytoprotection against Oxidative Stress by Methylnissolin-3-O-Β-D-Glucopyranoside from Astragalus Membranaceus Mainly Via the Activation of the Nrf2/Ho-1 Pathway. Molecules.

[23] Shaw R.J. (2009). LKB1 and AMP-Activated Protein Kinase Control of mTOR Signalling and Growth. Acta Physiol..

[24] Wu P., Liu S., Su J., Chen J., Li L., Zhang R., Chen T. (2017). Apoptosis Triggered by Isoquercitrin in Bladder Cancer Cells by Activating the AMPK-Activated Protein Kinase Pathway. Food Funct..

[25] Liu Y., Yang X., Gan J., Chen S., Xiao Z.-X., Cao Y. (2022). CB-Dock2: Improved Protein–Ligand Blind Docking by Integrating Cavity Detection, Docking and Homologous Template Fitting. Nucleic Acids Res..

